# Comment on “Bacteria in Cancer Therapy: Renaissance of an Old Concept”

**DOI:** 10.1155/2016/6304150

**Published:** 2016-09-14

**Authors:** Amin Talebi Bezmin Abadi

**Affiliations:** Department of Bacteriology, Faculty of Medical Sciences, Tarbiat Modares University, Tehran, Iran

Felgner et al. published an outstanding article reporting the bacteria as a novel approach to target cancer therapy to overcome current limitations of conventional therapies [1]. Lack of compliance to conventional anticancer treatments has prompted a call for alternative options [2, 3]. The possibility of using bacteria in cancer therapy has been determined many years ago [4]. At the moment, we do not recommend starting using this method as clinical solution for tumor or even other diseases. Taken together, some points we found may not support the proposed approach. It has been called a novel approach while still there are many pitfalls in application of bacteria because of their toxicity and deleterious side effects. For example, due to the intrinsic genetic instability of certain bacteria such as* Bifidobacterium longum* and* Escherichia coli*, there is no certainty about sufficient and consistent effect of this kind of therapy in long term of treatment. To date, we know that bacterial plasmid can be easily lost after a generation in human body; conclusively, our therapy can be quickly hampered. Notably, new genetic technology can help to reduce available problems but in close future.* In vivo* results had shown a relative success but more investigation regarding the targets that the bacteria should go for is necessary to provide a brilliant approach in cancer therapy. In other words, we are still in early ages to draw such an optimistic dream against various persistent tumors! Relatively high toxicity chance is the major difficulty with using bacteria as anticancer agents, a problem which needs different clinical trials with long-term follow-up. Notably, this problem can be even exacerbated if the used bacterium contains a potential to work as systemic microbe; then, occurrence of septicemia would be another obstacle. Last but not least, bacterial therapy will be even more expensive than other conventional treatments. As such, better suitable approaches should be recommended.

## References

[1] Felgner S., Kocijancic D., Frahm M., Weiss S. (2016). Bacteria in cancer therapy: renaissance of an old concept. *International Journal of Microbiology*.

[2] Jain K. K. (2001). Use of bacteria as anticancer agents. *Expert Opinion on Biological Therapy*.

[3] Abadi A. T. B. (2014). Therapy of *Helicobacter pylori*: present medley and future prospective. *BioMed Research International*.

[4] Lokody I. (2014). Anticancer therapy: bacterial treatment for cancer. *Nature Reviews Drug Discovery*.

